# Mesenchymal stem cells therapy for chronic ischemic stroke—a systematic review

**DOI:** 10.2478/abm-2024-0027

**Published:** 2024-10-31

**Authors:** Mohammad Kurniawan, Yetty Ramli, Nadira Deanda Putri, Salim Harris, Al Rasyid, Taufik Mesiano, Rakhmad Hidayat

**Affiliations:** Department of Neurology, Faculty of Medicine, University of Indonesia, Dr. Cipto Mangunkusumo National Hospital, Jakarta, Indonesia; Stem Cell Medical Technology Integrated Service Unit, Dr. Cipto Mangunkusumo National Hospital, Jakarta, Indonesia

**Keywords:** chronic, ischemic stroke, stroke, mesenchymal stem cells, treatment

## Abstract

Stroke represents a significant global health issue, primarily in the form of ischemic stroke. Despite the availability of therapeutic interventions, the recovery from chronic stroke, occurring 3 months post-initial stroke, poses substantial challenges. A promising avenue for post-acute stroke patients is mesenchymal stem cells (MSCs) therapy, which is derived from various sources and is globally recognized as the most utilized and extensively studied stem cell therapy. This systematic review, adhering to the Preferred Reporting Items for Systematic Reviews and Meta-Analysis (PRISMA) guidelines, aims to synthesize evidence regarding the impact of MSCs therapy on patients with chronic ischemic stroke. Employing an advanced search strategy across databases such as PubMed, PubMed Central, Google Scholar, the Cochrane Central Register of Controlled Trials (CENTRAL), and ClinicalTrial.gov, a total of 70 studies were identified, with 4studies meeting the inclusion criteria. Although positive outcomes were observed in terms of efficacy and safety, certain limitations, such as small sample sizes, study heterogeneity, and the absence of placebo groups, undermine the overall strength of the evidence. It is crucial to address these limitations in future research, highlighting the importance of larger sample sizes, standardized methodologies, and comparative trials to improve the assessment of MSCs’ efficacy and safety. Moving forward, key priorities include exploring underlying mechanisms, determining optimal administration modes and dosages, and conducting comparative trials. By addressing these aspects, we can propel MSCs therapies toward greater efficacy, safety, and applicability across diverse patient populations.

Stroke is the main contributor to the mortality and disability worldwide [1, 2]. Ischemic stroke, the most prevalent type, constituted 62.4% of all new cases in 2019. It occurs due to the obstruction of cerebral blood vessels, usually resulting from thrombosis or embolism [1, 3]. According to Cramer, chronic stroke refers to a stage occurring 3 months after the initial stroke when spontaneous recovery has plateaued, and the critical period for the recovery stage has concluded [4]. Current therapeutic approaches have shown limited effectiveness in providing substantial benefits to individuals with chronic stroke, particularly in terms of restoring impaired neural networks. As a result, there is an urgent demand for more effective options, and mesenchymal stem cells (MSCs) therapy emerges as a hopeful approach for improving outcomes in post-acute stroke patients. MSCs stand out as the most utilized and extensively studied stem cell therapy on a global scale [5, 6]. These cells can be extracted from bone marrow, adipose, dental pulp, and umbilical cord [7, 8]. Numerous preclinical studies have demonstrated its efficacy in chronic stroke, revealing recovery in rat models [9, 10]. However, examining ischemic stroke in an animal model proves impractical owing to the disease’s heterogeneous nature and complex pathophysiology [6]. To date, investigations into cell therapies for chronic stroke in humans have indicated satisfactory in safety and efficacy. Nevertheless, clinical trials have employed diverse methods, each with varying delays from the onset of the initial infarction [11]. Therefore, in this review, our aim is to consolidate the evidence regarding the impact of MSCs therapy on chronic stroke patients. Additionally, we will explore potential avenues for future research. By conducting this systematic review, our goal is to deepen our comprehension and actively contribute to endeavors focused on reducing the global burden of stroke.

## Methods

We followed the guidelines outlined in the Preferred Reporting Items for Systematic Reviews and Meta-Analyses (PRISMA) for the implementation of this systematic review. The procedure involved three primary stages: (1) conducting a thorough literature search, (2) extracting relevant data, and (3) critically appraising the chosen studies [12]. Additionally, we formulated research question using PICO structure, in which is phrased based on the components below:

*Population (P)*: Chronic ischemic stroke (3 months) patients.
*Intervention (I)*: MSCs therapy, regardless the source, route of administration, and dosage.
*Comparison (C)*: A group of patients who either received a placebo, underwent conventional stroke management, or had no comparison at all.
*Outcome (O)*: The main objective is to assess the outcomes with a minimum 6-month follow-up using the National Institutes of Health Stroke Scale (NIHSS), along with the Fugl–Meyer Assessment (FMA) and Bartell Index (BI). Both the modified and unmodified versions of FMA and BI are considered acceptable for evaluating effectiveness before and after implantation. The secondary outcome is to assess the safety and adverse events (AEs) related to the implantation procedures, which were assessed based on observed symptoms, including pain, seizure, headache, fever, death, neoplasm, myoclonus, dementia, ataxia, infection, vascular obstruction, and graft-versus-host disease, along with allergic reactions.

We conducted an advance search of published studies from the following databases: PubMed, PubMed Central, Google Scholar, the Cochrane Central Register of Controlled Trials (CENTRAL), and ClinicalTrial.gov (www.clinicaltrials.gov). These are the search strategies that we implemented in **Table 1**.

**Table 1.** Search keywords

**Electronic database name**	**Keywords**
PubMed	(Chronic[Title/Abstract]) AND ((Cerebral Ischemia[Title/Abstract]) OR (Ischemic Stroke[Title/Abstract]) OR (Middle Cerebral Artery Occlusion[Title/Abstract]) OR (Cerebral Infarction[Title/Abstract]) OR (Ischemic Brain Injury[Title/Abstract])) AND ((Bone Marrow Derived Stem Cells[Title/Abstract]) OR (Adipose Derived Stem Cells[Title/Abstract]) OR (Umbilical-Cord Derived Stem Cells[Title/Abstract]) OR (Mesenchymal Stem Cells[Title/Abstract]))
PubMed Central	(Chronic[Title/Abstract]) AND ((Cerebral Ischemia[Title/Abstract]) OR (Ischemic Stroke[Title/Abstract]) OR (Middle Cerebral Artery Occlusion[Title/Abstract]) OR (Cerebral Infarction[Title/Abstract]) OR (Ischemic Brain Injury[Title/Abstract])) AND ((Bone Marrow Derived Stem Cells[Title/Abstract]) OR (Adipose Derived Stem Cells[Title/Abstract]) OR (Umbilical-Cord Derived Stem Cells[Title/Abstract]) OR (Mesenchymal Stem Cells[Title/Abstract]))
Google Scholar	allintitle: mesenchymal stem cells chronic stroke
CENTRAL	“*Intervention* (‘Mesenchymal stromal cells’ OR ‘Bone marrow derived mesenchymal stromal cells’ OR ‘Umbilical cord mesenchymal stem cells’ OR ‘Human dental pulp mesenchymal stem cells’) AND *Population* (‘Ischemic Stroke’ OR ‘Occlusion Of Artery’ OR ‘Ischemic Stroke’ OR ‘Cerebral Infarction’)”
ClinicalTrials.gov	Condition “Chronic Ischemic Stroke” AND *Intervention* “Mesenchymal stem Cells”

CENTRAL, Cochrane Central Register of Controlled Trials.

Moreover, utilizing Research Rabbit, we examined the reference lists of the incorporated articles to identify pertinent studies. Additionally, we conducted a searched using term “mesenchymal stem cells chronic ischemic stroke” on Open Knowledge Maps. Following the retrieval of search results, we conducted a check for duplicates and screened the titles and abstracts. Subsequently, we imported the search results into the reference manager, specifically Mendeley. The criteria for data collection inclusion were as follows: (1) published in English, (2) studies conducted from 2011 to 2023, (3) studies focused on patients with chronic ischemic stroke, (4) done exclusively on human subjects, (5) clinical trial investigations encompassing both randomized and non-randomized designs, involving open-label, single-arm, and comparative studies, (6) studies utilizing MSCs for the treatment of chronic ischemic stroke, regardless of the cell source (allogeneic or autologous), method of administration (intravenous, intra-arterial, intrathecal, and intracerebral), and dosage, (7) the outcome measurements may include NIHSS, FMA, BI scores, or a combination of these assessments, and (8) minimum 6-month follow-up after the implantation. Excluded from consideration were publications in the form of commentaries, summaries, reviews, editorials, duplicates, or studies unrelated to the topic.

### Data extraction

Whenever feasible, we gathered information from the included studies, including data on the study’s author(s), publication year, country, number of samples, and duration from stroke to therapy. Furthermore, we extracted details concerning the intervention and control conditions, encompassing specific information about MSCs therapy, such as the cell source, route of administration, and dosage (if specified). Additionally, we documented the study outcomes, evaluated through NIHSS, FMA, and BI scores, as well as any observed AEs of MSCs, and noted the study limitations.

### Critical appraisal

We assessed the potential bias in the studies incorporated into our systematic review using the Newcastle-Ottawa Scale (NOS) [13]. This scale comprises three main components: selection (up to 4 points), comparability (up to 2 points), and outcome (up to 3 points). Studies scoring 5 or below were categorized as low quality, those scoring 6 or 7 were deemed of moderate quality, and studies with a score of 8 or 9 were considered high quality.

## Results

### Study selection

**Figure 1.**
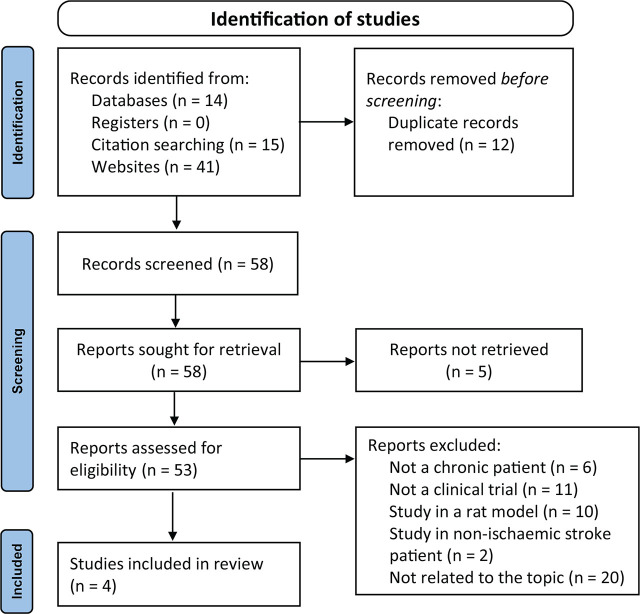
PRISMA flow diagram of the study selection process. PRISMA, Preferred Reporting Items for Systematic Reviews and Meta-Analysis.

A flowchart illustrating the study selection process is reported in **Figure 1**. Following our search strategies, a total of 70 articles were identified from electronic databases: 4 from PubMed, 1 from PubMed Central, 9 from Google Scholar, and none from CENTRAL and ClinicalTrials.gov. Besides that, we also retrieved 15 studies from Research Rabbit and 41 studies from Open Knowledge Maps. After a thorough quality appraisal, only 4 articles were included in this systematic review.

### Study characteristics

The breakdown of articles obtained as follows: three studies were sourced from PubMed [14,15,16] and one from Google Scholar [17]. The summarized characteristics of the studies can be found in **Table 2**. Four non-randomized studies, comprising a total of 69 samples, were included in the analysis. Only one study involved a comparison with a placebo group [18]. Two studies utilized allogeneic cells [15, 16], while two studies utilized autologous cells [14, 17]. Regarding the route of administration, two studies employed the intravenous method [15, 18], while the remaining two used stereotactic intracranial injection [14, 16]. All studies provided information on the dosage of cell therapy, ranging from 0.5 to 100 × 10^6^ across the board. Outcome measurements varied among the studies, with the majority including either NIHSS, FAM, BI, or all of them. Importantly, all studies had a follow-up period of at least 6-months post-therapy.

**Table 2.** Characteristics of extracted studies

**References**	**Location**	**Study design**	**Sample**	**Time from stroke to therapy**	**Cell source**	**Route of administration**	**Dosage**	**Outcome measure**	**Follow-up time**	**Comparison**	**NOS score**
Steinberg et al. [16]	USA	A non-randomized, open-label, single-arm study	18	Minimum 6 months	Allogeneic cells	Stereotactic intracranial injection	2.5 × 106, 5.0 × 106, or 10 × 106	ESS, NIHSS, FMA, F-M Motor Scale Score, mRS	On month 24	–	6
Levy et al. [15]	USA	A non-randomized, open-label, single-arm study	36	Minimum 6 months	Allogeneic cells	Intravenous	Phase I = 0.5 × 106, 1.0 × 106, and 1.5 × 106; phase 2 = 1.5 × 106/kg of body weight	NIHSS, BI, MMSE, Geriatric Depression Scale	On day 2, 3, 4, and 10, and on month 1, 3, 6, 9, and 12	–	6
Bhasin et al. [17]	India	A non-randomized, open-label study	MSCs group = 6; control group = 6	Minimum 3 months	Autologous cells	Intravenous	50–60 × 106	FMA, mBI	On week 8, 24, 78, 156, and 208	Placebo	7
Chiu et al. [14]	Taiwan	A non-randomized, open-label, single-arm study	3	Minimum 6 months	Autologous cells	Stereotactic intracranial injection	1.0 × 108	NIHSS, mFMA sensation, BI, Berg Balance Scale	On month 6	–	6

BI, Barthel Index; ESS, European Stroke Scale; FMA, Fugl–Meyer Assessment; mBI, modified Barthel Index; MMSE, Mini Mental State Examination; MSCs, mesenchymal stem cells; mRS, modified Rankin Scale; NIHSS, National Institutes of Health Stroke Scale; NOS, Newcastle-Ottawa Scale.

### Quality assessment

Using the NOS, we evaluated the risk of bias in each study, resulting in scores ranging from 6 to 7 stars, as written in **Table 2**. One study received a score of 7 stars [17], while the other received 6 stars [14,15,16]. Both scores categorize the studies as having a moderate risk of bias.

### Outcome assessment

#### Efficacy outcome

The combined findings from studies investigating the impact of MSCs interventions on stroke patients reveal positive outcomes. In the research conducted by Steinberg et al. [16] individuals with a baseline mean NIHSS total score of 9.3 ± 1.7 experienced significant improvements at 12 months (1.9) and 24 months (2.1). Similarly, the baseline mean FMA total of 133.6 ± 20.9 showed noteworthy improvements of 19.2 at 12 months and 19.4 at 24 months. Levy et al.’s [15] study, featuring an initial median NIHSS total score of 8 (interquartile range 6.5–10), demonstrated significant enhancements at 6 months (1) and 12 months (2). The baseline mean BI score of 65 ± 28.7 increased by 6.8 points at 6 months and 10.8 points at 12 months, with the percentage of patients achieving an excellent functional outcome (BI score 95) rising from 11.4% at baseline to 27.3% at 6 months and 35.5% at 12 months. Bhasin et al.’s [17] study indicated notable improvement in both intervention and control groups over 4 years, with only mBI exhibiting statistical significance when comparing the two groups at 208 weeks. Lastly, Chiu et al.’s [14] study highlighted significant improvements in motor and sensory functions at the 6-month mark.

#### Safety outcome

Across the four studies examining patients undergoing MSCs interventions, AEs were systematically monitored. In the study by Steinberg et al. [16] spanning 24 months, it was revealed that every patient in the cohort encountered at least one AE. Commonly observed AEs included headache related to surgical procedures, nausea, depression, muscle spasticity, vomiting, increased blood glucose, elevated C-reactive protein levels, constipation, fatigue, pain in extremity, and urinary tract infection. Most of these AEs were categorized as having mild or moderate intensity. In Levy et al.’s [15] study, a total of 15 serious AEs were documented, all assessed as unrelated or unlikely related to the investigational product. Two AEs, a urinary tract infection and intravenous site irritation, were potentially linked to the investigational product, both being mild and resulting in full recovery. Bhasin et al.’s [17] study reported one patient experiencing a skin allergy/rash 9 months post-transplantation, leading to hospitalization. However, it was determined that the infection was not related to the administered cells. Lastly, in Chiu et al.’s [14] study, AEs were reported, but the authors concluded they were unrelated to cell therapy, though specific details on these events were not provided. The efficacy and safety outcomes is summarized in **Table 3**.

**Table 3.** Summary of efficacy and safety outcomes

**References**	**Efficacy outcome**	**Safety outcome**	**Study limitation**
Steinberg et al. [16]	The mean ± SD of baseline NIHSS total score, 9.3 ± 1.7, significantly improved at 12 months (−1.9, 95% CI: −2.6 to −1.1, *P* < 0.001) and 24 months (−2.1, 95% CI: −3.3 to −1.0, *P* < 0.01). Similarly, the mean ± SD of baseline FM total score, 133.6 ± 20.9, showed significant improvement at 12 months (19.2, 95% CI: 11.4–27.0, *P* < 0.001) and 24 months (19.4, 95% CI: 9.9–29.0, *P* < 0.01).	At 24 months, all patients in the cohort experienced at least one AE. Common AEs and their respective percentages included headache related to surgical procedures (88.9%), nausea (33.3%), depression (22.2%), muscle spasticity (22.2%), vomiting (22.2%), increased blood glucose (16.7%), elevated C-reactive protein levels (16.7%), constipation (16.7%), fatigue (16.7%), pain in extremity (16.7%), and urinary tract infection (16.7%). Most of these AEs were mild (11.1%) or moderate (50.0%) in intensity.	Small sample size and the used of uncontrolled study design.
Levy et al. [15]	The median (interquartile range) of baseline NIHSS total score, 8 (6.5–10), significantly improved at 6 months (−1, *P* < 0.0001) and 12 months (−2, *P* < 0.001). The mean ± SD of baseline BI score, 65 ± 28.7, showed a notable gain of 6.8 points at 6 months (*P* = 0.0002) and further escalated to 10.8 points at 12 months (*P* < 0.001). The percentage of patients achieving an excellent functional outcome (BI score ≥95) increased from 11.4% at baseline to 27.3% at 6 months and 35.5% at 12 months.	Out of 15 serious AEs, all were deemed unrelated or unlikely related to the investigational product. Among the 109 reported AEs, two were possibly related: a mild urinary tract infection and mild intravenous site irritation, both resulting in full recovery. Additional details are available in the study report.	There was no control group and no study regarding the mechanism of action.
Bhasin et al. [17]	In the MSCs group, both FM and mBI scores increased from baseline to the 208th week. The control group also showed notable improvement from baseline to the 4-year assessment. Baseline characteristics were aligned (*P* > 0.05), confirming comparability for evaluating therapy effectiveness at 8, 24, 78, 156, and 208 weeks. Notably, only mBI exhibited statistical significance at 208 weeks (95% CI: −12.9 to 0.49, *P* = 0.05) and at 156 weeks (95% CI: −1.26 to 1.76, *P* = 0.04). No significant difference in FM scores was observed at the 4-year examination (95% CI: −3.01 to 2.01, *P* = 0.19).	After 9 months post-transplantation, one patient reported a skin allergy/rash. Subsequently, the patient was hospitalized for the issue, but it was determined that the infection was not related to the administered cells.	Small sample size.
Chiu et al. [14]	At 6 months, all patients exhibited improved motor functions (2–5 points) and sensory functions (0–2 points) compared to the initial NIHSS score. FMA scores indicated enhancements in both motor and sensory functions, while BI results highlighted improvements in daily life activities.	AEs were reported, yet the authors concluded that they were unrelated to the administration of cell therapy. Unfortunately, specific details about these AEs were not provided.	The sample size is limited and there is no group for comparison.

AEs, adverse events; BI, Bartell Index; FMA, Fugl–Meyer Assessment; MSCs, mesenchymal stem cells; NIHSS, National Institutes of Health Stroke Scale.

## Discussion

Stem cells have played a significant role in modern regenerative medicine since the 1950s, notably with the occurrence of the first bone marrow transplantation in 1956. This milestone not only showcased the immediate possibilities but also paved the way for future developments and advancements in clinical techniques, laying the foundation for the stem cell therapies we have today [19, 20]. Presently, stem cell therapies are recognized as potential neuroregenerative interventions, applicable not only to acute stroke patients but also to those in the chronic phase [21, 22]. The initial human investigations into cell therapy for chronic stroke can be traced back to the early 2000s [23, 24]. According to a meta-analysis by Chen et al. [25] stem cell-based therapy shows promise in improving impaired functions and enhancing the quality of life in stroke patients. Nonetheless, the current landscape of stem cell therapies presents notable challenges, including a restricted supply of engraftable stem cells, the necessity for an optimal treatment timeframe, inherent limitations in the potential of adult stem cells, and potential adverse effects linked to transplanted cells, such as tumor formation or stroke [26]. This study specifically aimed to evaluate the effectiveness and safety of MSCs interventions in individuals with chronic ischemic stroke. The systematic review encompassed four eligible studies, emphasizing the limited availability of research on this subject. It is essential to recognize the scarcity of studies in this domain, and the existing data may not fully meet optimal quality standards. Despite these limitations, this paper endeavors to delve into key insights derived from the chosen articles, offering valuable considerations to guide future research endeavors into MSCs interventions for individuals with chronic ischemic stroke.

### MSCs interventions for chronic ischemic stroke: efficacy and safety

The rising global impact of stroke necessitates urgent exploration of effective treatments to improve clinical outcomes and reduce the overall burden. When an infarct progresses from the acute to the chronic phase, neural tissue is almost entirely lost, resulting in significant challenges including: (1) neuronal loss, (2) glial scar formation (3) presence of reactive astrocytes, (4) activation of microglia/macrophages, and (5) disrupted blood supply [27]. Improvements in outcomes are likely due to mechanisms such as the release of growth factors, anti-inflammatory effects, and indirect effects mediated by local tissue remodeling [28]. MSCs have emerged as a promising treatment for chronic ischemic stroke patients. Presently, MSCs are extensively studied and employed in clinical trials for treating various diseases [29, 30].

A review of four non-randomized studies involving 69 participants, with only one study including a placebo group, indicated the safety and positive clinical outcomes associated with MSC interventions in chronic ischemic stroke patients. Most AEs during trials were mild to moderate and were often unrelated to MSC administration. The studies used both allogeneic and autologous MSCs, administered either intravenously or via stereotactic intracranial injection. Vahidy et al. [31] noted that intravenous administration is safe and easy to perform, but suggested that stem cells might be cleared in the bloodstream and may not cross the blood-brain barrier, requiring a large quantity of cells [6]. Conversely, intracranial injection can deliver cells directly to the target location with a smaller cell dose but is an invasive procedure [6].

**Figure 2.**
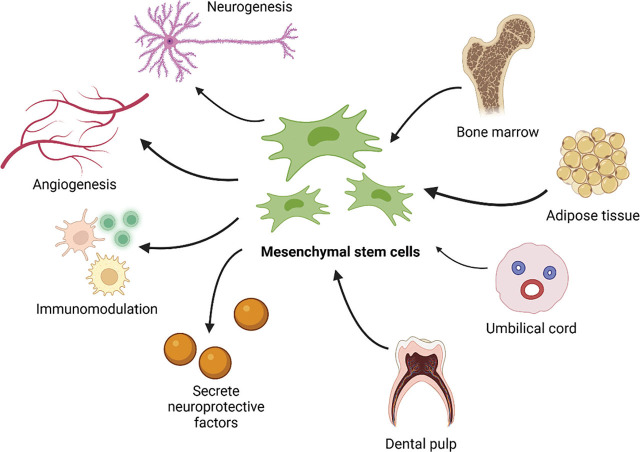
MSCs can be sourced from bone marrow, adipose tissue, umbilical cord, and dental pulp. These cells can treat ischemic stroke by secreting neuroprotective factors, acting as immunomodulators, and promoting angiogenesis and neurogenesis ^[32,33,34]^. Created with BioRender.com. MSCs, mesenchymal stem cells.

Several studies have demonstrated the therapeutic potential of MSCs in treating stroke patients. MSCs operate through various mechanisms, including promoting angiogenesis, neurogenesis, synaptogenesis, immunomodulation, paracrine effects, neuroprotection, and reducing oxidative stress [32,33,34]. MSCs promote angiogenesis by increasing levels of angiogenesis-related proteins such as angiogenin-1 (Ang1), tyrosine-protein kinase receptor (Tie2), VEGF, and VEGF receptor 2 (Flk1), which enhance blood vessel density at the site of vascular injury [35]. Additionally, MSCs enhance neurogenesis by boosting axonal growth-associated proteins and reducing axonal growth-inhibiting proteins, thereby promoting axonal growth [36]. They also help decrease blood-brain barrier disruption and neuronal loss by increasing collagen IV and tight junction protein ZO-1 [37, 38]. The sources and therapetic mechanisms of MSCs is depicted in **Figure 2**.

Jingli et al. [32] proposed three key hypotheses regarding the mechanisms of MSCs:

(1) MSCs may modulate the immune system to inhibit damaging autoreactive responses and protect the central nervous system.
(2) MSCs can secrete neuroprotective factors such as glial cell line-derived neurotrophic factor and brain-derived neurotrophic factor.
(3) MSCs might have the potential to transdifferentiate, leading to tissue renewal, although this hypothesis is debated among researchers. MSC transplantation may contribute to brain tissue regeneration by differentiating into neurons and glial cells under appropriate conditions [32, 39].

Chiu et al. [14] suggest that MSC implantation can regulate hematopoiesis, promote localized angiogenesis by recruiting endothelial progenitor cells, and attract bone marrow-derived cells and neural progenitor cells (NPCs) to the injury site. This aligns with findings by Shiota et al. [40] who observed that MSC transplantation significantly increased the proliferation, migration, and differentiation of endogenous NPCs in cerebral ischemic conditions.

### Strengths and limitations

This systematic review represents a groundbreaking exploration of the efficacy and safety of MSC interventions for chronic ischemic stroke patients, marking the first of its kind in this domain. While this pioneering aspect significantly contributes to our understanding, the study is not without limitations. The relatively small sample size across included studies is a notable constraint, potentially impacting the generalizability of findings and underscoring the necessity for larger-scale investigations. Additionally, the absence of multiple studies featuring a placebo group is a substantial limitation, reducing the overall strength of evidence regarding the effectiveness and safety of MSC interventions. The observed high heterogeneity among studies, characterized by variations in therapeutic characteristics like administration route, timing post-stroke, and dosage, poses a challenge and may introduce confounding factors, complicating result interpretation. Lastly, the study’s restriction to English publications introduces the potential for language bias, possibly excluding valuable data from non-English sources and limiting the review’s comprehensiveness. Future research endeavors should address these limitations to enhance the robustness and applicability of findings in the realm of stem cell interventions for stroke.

### Future directions

Although studies on MSC-based therapy for stroke have shown promising results, several challenges must be addressed before MSCs can be widely used in clinical practice. The optimal route of administration, dosage, and source of MSCs for treating chronic ischemic stroke has yet to be determined. Therefore, more research is essential to advance our understanding and application of this promising therapy.

Firstly, future research should prioritize increasing sample sizes and conducting trials with comparative groups. Expanding the diversity and size of study populations will provide a more comprehensive evaluation of MSCs’ efficacy and safety in chronic ischemic stroke. Comparative trials with control groups will enable thorough assessments, helping researchers identify the specific benefits of MSC interventions and establish their superiority over existing or alternative treatments. Secondly, establishing a standardized methodology is crucial to ensure the reproducibility of MSC interventions across different populations. Standardized protocols will facilitate consistent application and reliable results. Thirdly, exploring the intricate mechanisms underlying MSC treatment for chronic ischemic stroke is essential. Investigating the molecular and cellular processes involved will deepen our understanding of how MSCs facilitate recovery and guide the development of targeted interventions. Lastly, future research should focus on determining the optimal mode of administration and dosage. Examining various administration routes and dosage regimens will help identify the most effective and patient-friendly approaches. Comprehensive insights into these aspects are vital for optimizing treatment outcomes and minimizing potential side effects.

## Conclusion

MSCs interventions for chronic ischemic stroke demonstrate favorable results in enhancing both neurological and functional aspects, accompanied by a positive safety profile. However, limitations in sample size, study heterogeneity, and the absence of placebo groups impact the strength of evidence. Addressing these limitations is crucial for future research. Prioritizing larger sample sizes, standardized methodologies, and comparative trials will enhance the assessment of MSCs efficacy and safety. Exploring underlying mechanisms and determining optimal administration modes and dosages are key areas for refining MSCs interventions. By addressing the outlined research priorities, the scientific community can contribute to the ongoing evolution of MSCs therapies, ultimately enhancing their efficacy, safety, and applicability for a broader spectrum of patients.
